# Controlling of lattice strains for crack-free and strong ferroelectric barium titanate films by post-thermal treatment

**DOI:** 10.1038/s41598-022-09182-0

**Published:** 2022-03-30

**Authors:** Bogyu Kim, Young-Uk Jeon, Chulwoo Lee, In Soo Kim, Byeong-Hyeon Lee, Young-Hwan Kim, Young Duck Kim, Il Ki Han, Kwanil Lee, Jongbum Kim, JoonHyun Kang

**Affiliations:** 1 Nanophotonics Research Center, Korea Institute of Science and Technology (KIST), Seoul, 02792 Republic of Korea; 2 KHU-KIST Department of Converging Science and Technology, Kyung Hee University, Seoul, 02447 Republic of Korea; 3 School of Electrical Engineering, Korea University, Seoul, 02841 Republic of Korea; 4 KIST-SKKU Carbon-Neutral Research Center, Sungkyunkwan University (SKKU), Suwon, 16419 Republic of Korea; 5 Advanced Analysis Center, Korea Institute of Science and Technology (KIST), Seoul, 02792 Republic of Korea; 6 Department of Physics and Department of Information Display, Kyung Hee University, Seoul, 02447 Republic of Korea

**Keywords:** Ferroelectrics and multiferroics, Surfaces, interfaces and thin films, Nonlinear optics, Nonlinear optics

## Abstract

In this study, we experimentally demonstrate fabrication of ultra-smooth and crystalline barium titanate (BTO) films on magnesium oxide (MgO) substrates by engineering lattice strain and crystal structure via thermal treatment. We observe that oxygen-depleted deposition allows growth of highly strained BTO films on MgO substrates with crack-free surface. In addition, post-thermal treatment relaxes strain, resulting in an enhancement of ferroelectricity. Surface roughening of the BTO films caused by recrystallization during post-thermal treatment is controlled by chemical–mechanical polishing (CMP) to retain their initial ultra-smooth surfaces. From Raman spectroscopy, reciprocal space map (RSM), and capacitance–voltage (C–V) curve measurements, we confirm that the ferroelectricity of BTO films strongly depend on the relaxation of lattice strain and the phase transition from *a*-axis to *c*-axis oriented crystal structure.

## Introduction

Ferroelectricity is a key property of perovskite materials exhibiting spontaneous electric polarization, which can alter the physical properties of materials by the application of an external electrical field. In particular, barium titanate (BTO) has been widely studied as a promising perovskite-type ferroelectric and piezoelectric material for the development of diverse optical and electrical devices, including capacitors^1^, infrared detectors^2^, and non-volatile memory^3,4^. Especially, recent advances in BTO thin films for the development of high-speed and low-power consumption electro-optic (EO) modulators have attracted much attention for various types of photonic integrated circuits mostly in the near infrared range, taking advantage of the high Pockels effect^5–9^ which the refractive index of materials can be modified in proportion to the applied electric field. Considering superior EO properties of BTO films on Si substrates, it is necessary to explore the growth of BTO films on transparent substrates to extend the operating wavelength of EO devices to the visible spectral range.

Among several transparent oxide substrates, magnesium oxide (MgO) has been considered as the most suitable substrate for on-chip waveguides and modulators because of its low optical index ($n= \sim 1.73$)^10^, which satisfies wave-guiding conditions when a BTO film is directly deposited on the MgO substrate. However, it is challenging to grow highly-crystalline BTO (${a}_{BTO}= \sim 3.996\, {\AA }, {c}_{BTO}= \sim 4.029 \,{\AA }$) film on MgO (${a}_{MgO}= \sim 4.213 \,{\AA }$) because of the lattice mismatch (lower than 5.4%)^11^, which causes large strain on the film and roughens its surface. The buffer layer such as strontium titanate (STO) has been utilized to reduce the lattice strain in BTO films, but the growth of epitaxial buffer layer is also a demanding process^12,13^. In this paper, we will present a simple but versatile method to fabricate crack-free, ultra-smooth and strong ferroelectric BTO films on MgO by controlling the lattice strain through optimizing oxygen partial pressure during deposition and post-thermal treatment without the support of any buffer layer.

**Figure 1:**
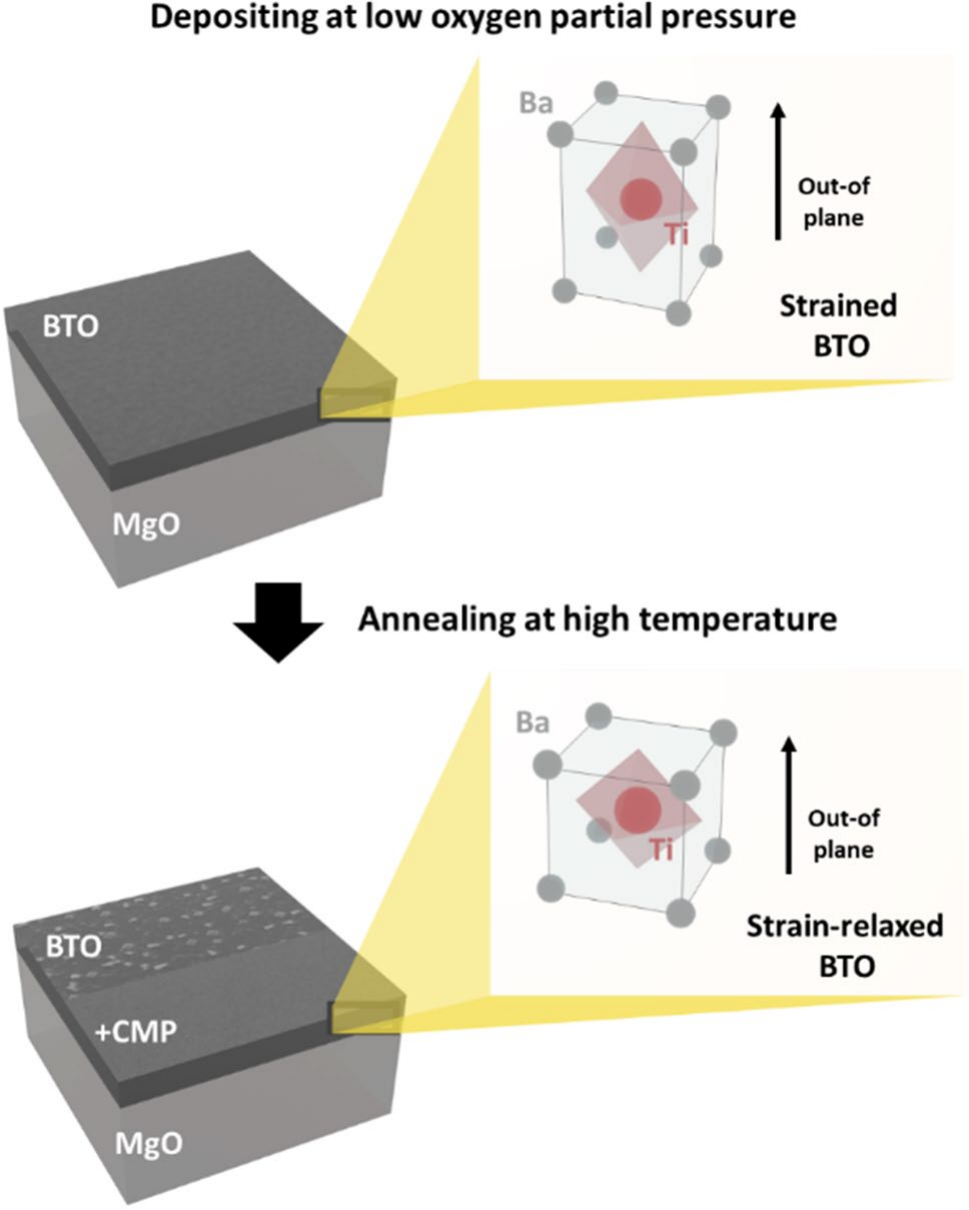
Schematic image of the fabrication of ultra-smooth and strong ferroelectric BTO film by controlling lattice strains.

The significance of substrate induced strain in BTO film has been highlighted by its potential to enhancement of ferroelectric properties, the control of Curie temperature, and the formation of nanostructures^14–16^. Therefore, strain engineering is remarkably critical to improve the device performance. Well-engineered strain through proper selection of substrate can greatly enhance certain properties of a film^14^, but such strain may also lead to the degradation of film properties when the choice of suitable substrate is limited. Since the device performance depends not only on the physical properties of films but also on their surface, it is very important to utilize strain to grow crack-free and ultra-smooth films, especially in the case of EO applications where the waveguide is required for optical signal propagation. As depicted in Fig. 1, we first optimize the oxygen partial pressure for the growth of crack-free highly-strained BTO films on MgO substrates using pulsed laser deposition (PLD). The as-deposited films are then annealed to enhance the ferroelectric characteristics while maintaining the crack-free surface. The annealed BTO films are polished to retain the surface roughness and smoothen the textured surface caused by the growth of unexpected grains during post-annealing. Based on these processes, we successfully fabricate a 500 nm thick BTO film with a 0.23 nm root mean square (RMS) roughness, which is sufficient to realize an EO modulator with a waveguide on its surface.

## Materials and methods

### Film deposition

The BTO films were deposited on MgO substrates with (001) orientation by PLD using a KrF excimer laser (Coherent, Inc.) operating at a wavelength of 248 nm for source material ablation. BTO ceramic target was purchased from Toshima Co., Ltd. with a purity of 99.99% or higher. The substrate temperature during the BTO film deposition was maintained at 700 °C, and the repetition rate and energy density of the laser beam were set to 5 Hz and 1.4 J/cm^2^, respectively. The oxygen partial pressure (P_oxygen_) was varied between 10 and 200 mTorr to optimize the surface roughness.

### Film characterization

The polarized Raman spectra of the BTO films were recorded in backscattering geometry at room temperature (25 °C) using a microscope and a 100 × objective. The 532 nm line of a diode-pumped solid-state laser was used for excitation with a spot diameter of 1 μm. The scattered light was analyzed using a spectrometer equipped with a charge-coupled device (CCD) detector (iDUS DV401A-BV-352, Andor Technology Plc) in the Stokes frequency range of 200–900 cm^−1^. High-resolution X-ray diffraction (HRXRD) measurements were performed using a Rigaku ATX-G system with monochromated CuKα1 $(\lambda =1.5406 \,{\AA})$. Detailed information about the domain structure was ascertained from the reciprocal space map (RSM) of the 224 diffraction maxima of the BTO films. Atomic force microscopy (AFM; XE-100, Park Systems) was used to obtain the surface roughness of the BTO films.

### Post processing of thin films

The BTO films were annealed in an electric furnace (Carbolite CWF 1200, Carbolite) at temperatures of 750, 900, 1050, and 1150 °C, which are higher than the deposition temperature. The annealing was performed in air and the desired annealing temperature (T_annealing_) was maintained for 30 min. The annealed BTO films were then cooled to room temperature at a rate of 5 °C/min to prevent cracking. A 20 nm colloidal-silica-based chemical mechanical polishing (CMP) was performed to achieve an ultra-smooth film from the textured BTO films produced by annealing.

### C–V characterization

The C–V curve was characterized using an LCR meter (4284A, Hewlett Packard) to quantitatively analyze the ferroelectricity of the BTO film. To study the effect of annealing on the ferroelectric characteristics, small signal C–V measurements were conducted for the BTO thin film before and after annealing. After polishing the surface of the BTO thin films on the top, 2-µm-spaced and 3-mm-long Cr/Au electrodes were patterned at different angles. Using these 2-µm-spaced electrodes, the C–V curve was measured by applying a voltage between − 20 and 20 V at 10 kHz.

## Results

**Figure 2:**
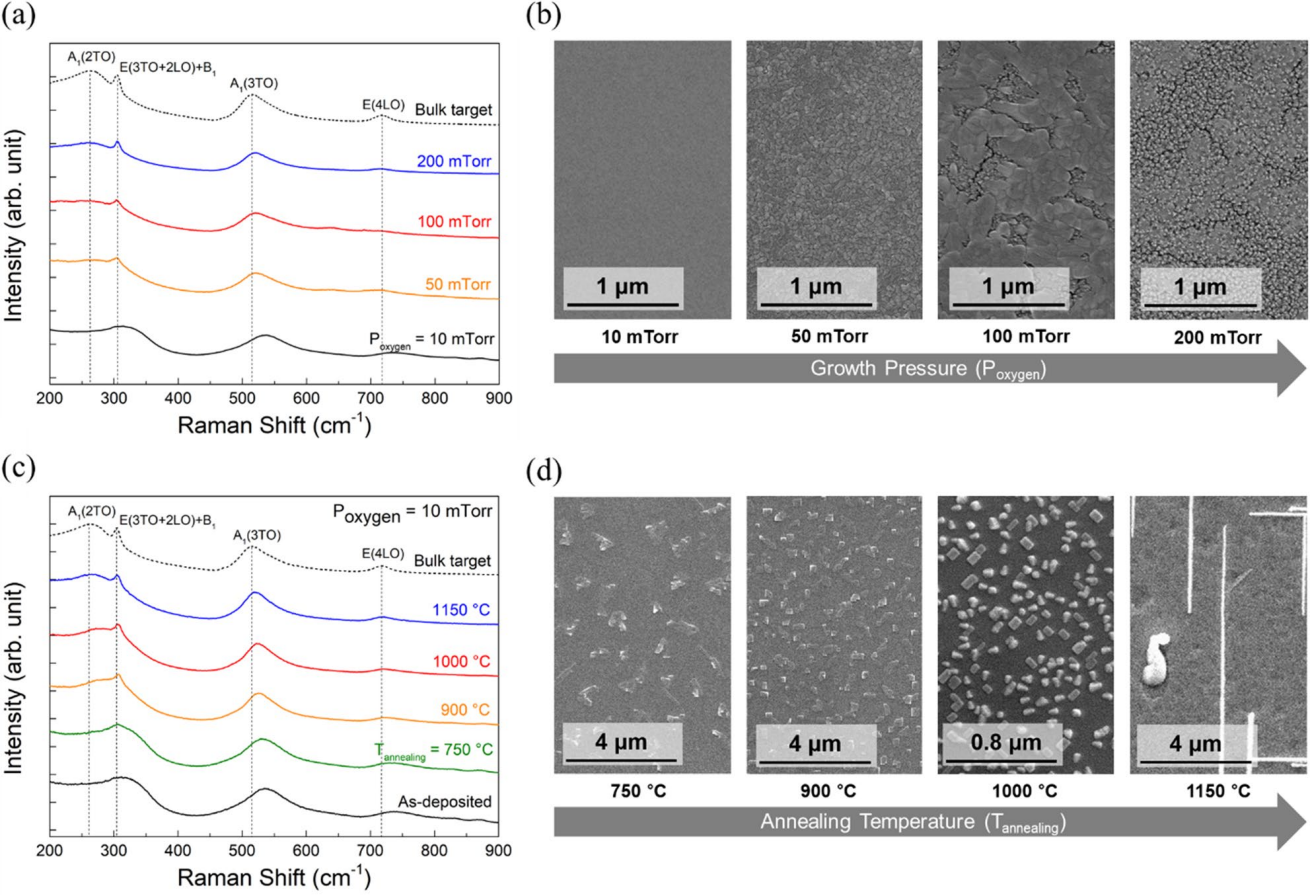
(**a**) Raman spectra of BTO bulk target and BTO films deposited under different oxygen partial pressures (P_oxygen_; 10, 50, 100, and 200 mTorr). (**b**) Scanning electron microscopy (SEM) images of BTO films for each oxygen partial pressure. (**c**) Raman spectra of BTO bulk target, as-deposited BTO film, and BTO films annealed at 750, 900, 1000, and 1150 °C. (**d**) SEM images of BTO films annealed at 750, 900, 1000, and 1150 °C. SEM image of BTO film annealed at 1000 °C has a higher magnification to clearly display nanoscale particles with the size of a few to several nanometers.

Figure 2(a) shows the Raman spectra of the BTO target and BTO films grown at different oxygen partial pressures. The Raman spectra of the polycrystalline bulk target consists of several peaks centered at 260 cm^−1^, 305 cm^−1^, 519 cm^−1^, and 720 cm^−1^, which corresponds to A1 (2TO), E (3TO + 2LO) + B1, A1 (3TO), and E (4LO) phonon modes, respectively^17,18^. In particular, a comparison of E phonon soft modes (3TO + 2LO, 4LO) between the film and bulk target provides valuable information on the ferroelectricity of BTO, because these Raman bands are induced by the spontaneous polarization (P_s_) of incoming light^19^. When the BTO film is grown at a low oxygen partial pressure of 10 mTorr, the Raman bands of A1 (2TO), A1 (3TO), and E (4LO) phonon modes exhibit stokes shifts from the those of the BTO target, and no peak at 305 cm^−1^ (E (3TO + 2LO) + B1) is observed. With increasing oxygen partial pressure, the 305 cm^−1^ peak becomes stronger and the Raman bands of the films gradually align with the peaks of the BTO target, indicating an enhancement of ferroelectricity. However, as shown in Fig. 2(b), high oxygen partial pressures induce increased surface roughness with formation of cracks, which can seriously degrade the performance of on-chip waveguides due to light scattering.

Instead of enhancing the ferroelectricity of BTO films by depositing at high oxygen partial pressure, post-annealing was adopted to control the ferroelectricity of the BTO films. To achieve crack-free BTO films, the BTO films were prepared at low oxygen partial pressure of 10 mTorr prior to the post-annealing process. Figure 2(c) shows the Raman spectra of the BTO target and BTO films annealed at different temperatures. Similar to increasing the oxygen partial pressure during deposition, the Raman bands of the films gradually align with the peaks of the BTO target, and the 305 cm^−1^ peak becomes stronger with increasing annealing temperature. Variations in the surface morphology due to the annealing was also monitored, as shown in Fig. 2(d). With increasing annealing temperature, randomly distributed rectangular features were formed; needle-like structures were observed on the surface of the BTO films annealed at 1150°C^20^. Nevertheless, films do not suffer from cracking even when the ferroelectricity of the films is enhanced by annealing.

**Figure 3:**
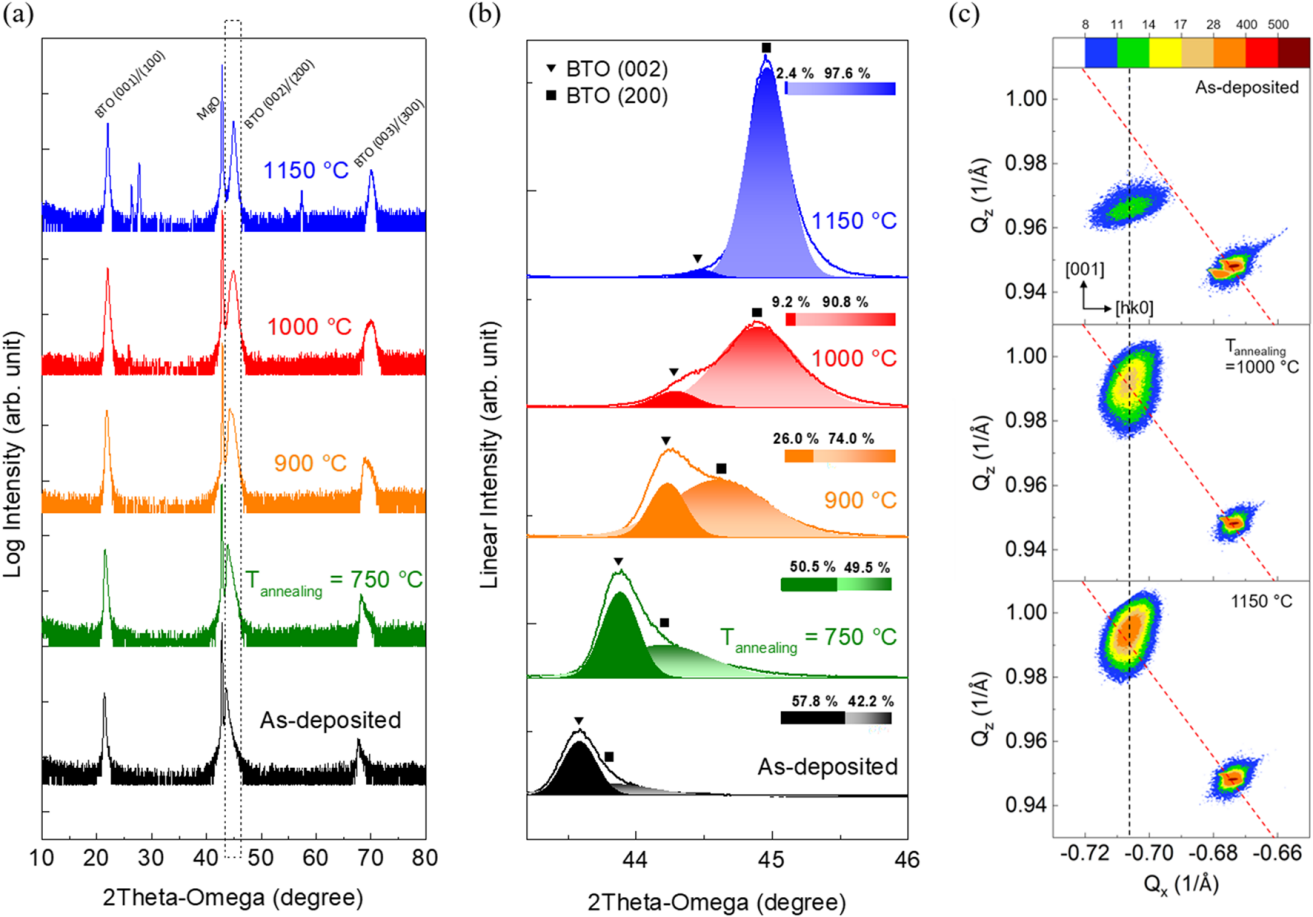
(**a**) X-ray diffraction (XRD) patterns of as-deposited BTO film and BTO films annealed at 750, 900, 1000, and 1150 °C. (**b**) X-ray diffraction 2θ-ω scan of the BTO (002)/(200) peaks (the area in the dashed box in (**a**)). Solid line indicates the experimental XRD spectrum, and the combined XRD peak is analyzed as the sum of two Gaussian curves. The triangle mark and the square mark represent the position of (002) and (200) peak, respectively. The composition ratios of each pick are expressed as a ratio of area. (**c**) Reciprocal space mapping (RSM) of the 224 diffraction of as-deposited BTO film and BTO films annealed at 1000, 1150 °C. The color bar represents the intensity of the measured signal. Red dashed line corresponds to the condition of strain relaxation. The black dashed line is parallel to the [001] direction.

To determine the influence of annealing on the crystallinity, HRXRD measurements were performed. Figure 3(a) shows the out-of-plane XRD pattern displaying the change in the crystal structure by post-annealing. The diffraction patterns were analyzed using the reference pattern of the tetragonal BaTiO_3_ (JCPDS no. 04-014-0448). As the annealing temperature increased, the intensity of the XRD peaks from the (001) plane increased, and some additional peaks were observed for the samples annealed at 1000 and 1150 °C. Upon closer inspection of the (002) peak as shown in Fig. 3(b), the (002) peak of the as-deposited film is located near the MgO (002) peak, and the (002) peak of the annealed films is shifted from 43.6 to 45.0° with increasing annealing temperature. Strikingly, we observe that the BTO 002/200 peak broadens with the evolution of two components located between (200) and (002) diffractions of the bulk BTO. The tetragonal distortion between a- and c-axis oriented domains is an important factor to determine the ferroelectricity of materials. Therefore, several groups^9,21^ presented that the splitting of XRD peaks of BTO films is attributed by the presence of poly-domain structure consisting of both *c*-axis (002) and *a*-axis (200) oriented domains. The evolution of the two components upon annealing demonstrates the transformation of the dominantly c-axis oriented tetragonal structure in the as-deposited films to the dominantly a-axis oriented tetragonal structure in the annealed films.

For further analysis of the change in the orientation of crystal domains, RSM measurements at the 224 diffraction maxima of the BTO films were performed as shown in Fig. 3(c). RSM images of the as-grown and annealed BTO films provide a clear explanation of the recrystallization behavior in the BTO films. The RSM of the as-deposited BTO film shows that the diffraction peak position of the BTO film is located at a distance from the relaxation line (red dashed line), and this peak migrates along the vertical line toward the red dashed line with increasing annealing temperature. This result indicates that the post-annealing only induces a change in the out-of-plane lattice constant while preserving the in-plane lattice constant. In other words, the as-deposited BTO film at lower oxygen partial pressure is strongly strained along the *c*-axis and the post-annealing step helps to relieve out-of-plane strain with minimal impact on the strain along the in-plane orientation. Therefore, we can conclude that the transformation of domain structure is mainly attributed by the strain relaxation along the *c*-axis.

**Figure 4:**
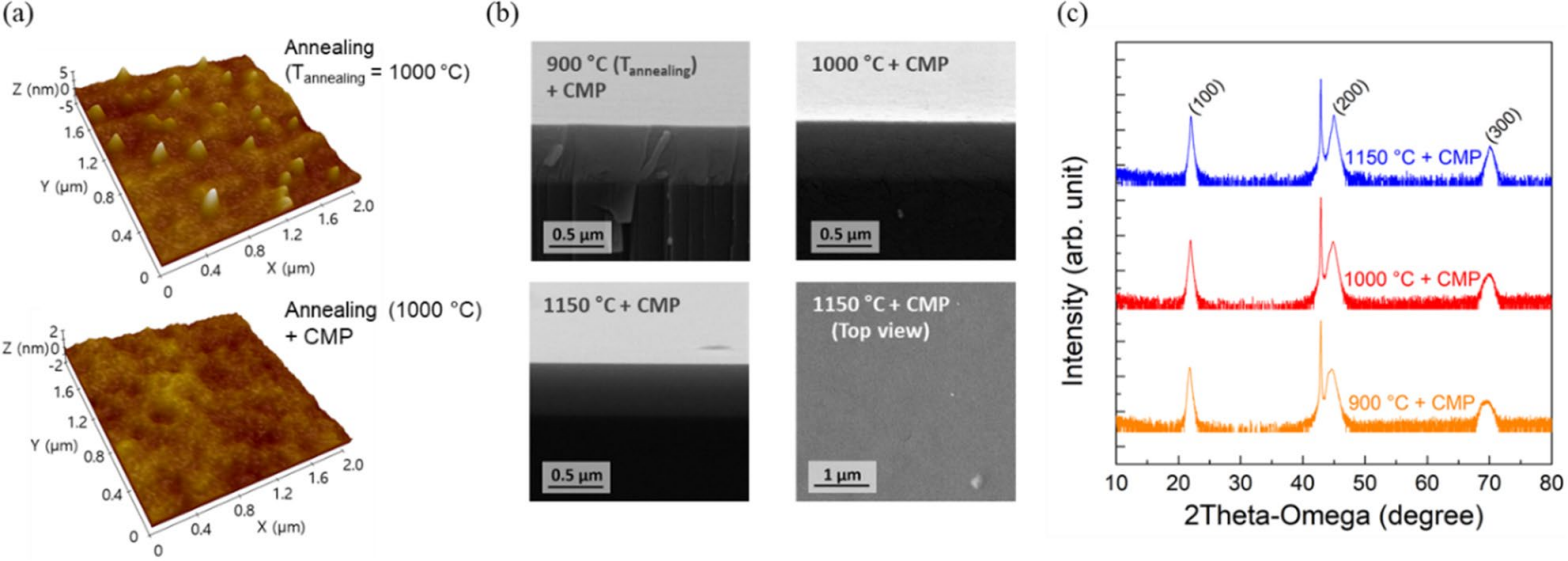
(**a**) Three-dimensional atomic force microscopy (AFM) images of BTO films annealed at 1000 °C before and after CMP. Z-axis scale of the film before CMP is larger than that of the film after CMP because of surface textures. (**b**) Cross-sectional scanning electron microscopy (SEM) images of BTO films annealed at 900, 1000, and 1150 °C after CMP, and the top view of the SEM image of the BTO film annealed at 1150 °C after CMP. (**c**) XRD patterns of BTO films annealed at 900, 1000, and 1150 °C after CMP.

To retain ultra-smooth surface of the BTO films, we performed a CMP process as shown in Fig. 4(a). We observe that the RMS roughness of BTO film annealed at 1000 °C was reduced from 1.4 nm to 0.23 nm after the CMP process. Furthermore, cross-sectional SEM images of the polished BTO films (Fig. 4(b)) clearly show a smooth, uniform, and texture-free surface after the CMP in BTO films annealed at 900 and 1000 °C. However, the textured surface with sharp nanoscale features of the BTO film annealed at 1150 °C were not completely removed by the CMP. A two-step CMP process using different sizes of colloidal-silica for coarse and fine polishing, effectively eliminates nanoscale features. However, the two-step CMP was not performed in this work in order to minimize the influence of thickness on the ferroelectric properties obtained by C-V measurements. Additionally, the XRD spectra obtained after CMP (Fig. 4(c)) demonstrates that additional XRD peaks induced by the annealing originate from surface micro-texturing because all extra peaks disappear after the polishing.

**Figure 5:**
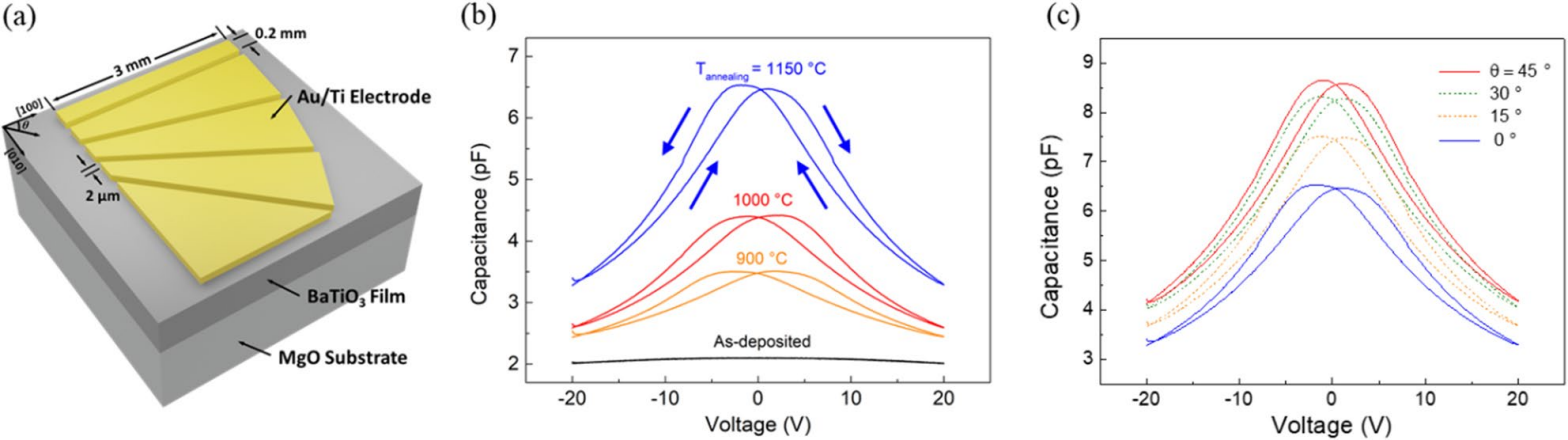
(**a**) Schematic image of Au/Ti coplanar electrodes on BTO film. Electrode with *θ* = 0° was aligned to MgO [100] direction and each channel between electrodes was aligned at 0, 15, 30, and 45° to MgO [100] direction. (**b**) Capacitance–voltage (C–V) curves of BTO film at different post-annealing temperatures (T_annealing_; 900, 1000, and 1150 °C) measured with a coplanar electrode parallel to the MgO [100] direction. The direction of voltage sweep (indicated by arrows) is from −20 to 20 V and then from 20 to −20 V. (**c**) Capacitance–voltage curves of BTO film annealed at 1150 °C with electrode angles (*θ*) of 0, 15, 30 and 45°.

For C–V measurements, electrodes were patterned on the film surfaces as shown in Fig. 5(a). The 2-µm-spaced channel between two electrodes is designed to align with BTO crystal orientation axis. The electrode pattern consists of four channels at angles of 0, 15, 30, and 45° with respect to the [100] direction of the BTO film. We have determined the crystal orientation of BTO films by polarized Raman measurements; the intensity of Raman scattering was at its minimum when the electric field of incident light was aligned along the [100] direction of MgO substrate. Figure 5(b) shows the C–V characteristics of the BTO films measured at a frequency of 10 kHz, in which the electrodes are aligned along the MgO [100] direction. Interestingly, the BTO film without annealing showed no signs of ferroelectric characteristics with a low dielectric constant. On the other hand, BTO films annealed at temperatures of up to 900 °C clearly show butterfly loops with large capacitances and lower coercive voltages. In addition to the C–V characteristics of the annealed films, the angular dependence of ferroelectricity due to the tensorial nature of electro-optic effects were measured from the channels aligned at angles of 15, 30, and 45° with respect to the [100] direction of BTO film annealed at 1150 °C. As displayed in Fig. 5(c), the maximum capacitance and minimum coercive voltage were measured at an angle of 45°. The largest capacitance value at 45° confirms the randomly distributed *a*-axis oriented tetragonal domains. The estimated coercive voltage extracted from the sample aligned at 45° was Ec = 7.25 × 10^5^ V/m, which is much smaller than the previously reported coercive voltage of the BTO films deposited by PLD method^22^.

## Conclusions

We demonstrate a unique approach to fabricate crack-free, ultra-smooth and strong ferroelectric BTO films by modifying the domain structure and lattice strain. Highly strained BTO films deposited at low oxygen partial pressure do not exhibit ferroelectricity, but no crack is observed on the film surface. The ferroelectricity of BTO films deposited at low oxygen partial pressure can be enhanced by thermal treatment. The XRD and RSM measurements confirmed that the post-annealing relaxes out-of-plane lattice strain and induces transformation of *a*-axis oriented domains into *c*-axis oriented domains. Lastly, the C–V curve measurements verify the enhancement of the ferroelectric characteristic and the tensor nature of ferroelectricity of the annealed BTO films. Our experimental results on controlling lattice strain to fabricate crack-free and ultra-smooth crystalline BTO film with high ferroelectric properties may provide a simple route toward improving the performance of BTO-based optical on-chip devices and electrical devices.
